# Neurogenesis of Subventricular Zone Progenitors in the Premature Cortex of Ferrets Facilitated by Neonatal Valproic Acid Exposure

**DOI:** 10.3390/ijms23094882

**Published:** 2022-04-28

**Authors:** Kazuhiko Sawada

**Affiliations:** Department of Nutrition, Faculty of Medical and Health Sciences, Tsukuba International University, Tsuchiura 300-0051, Ibaraki, Japan; k-sawada@tius.ac.jp; Tel.: +81-29-883-6032

**Keywords:** valproic acid, outer subventricular zone, basal radial glia, Pax6, ferret

## Abstract

The present study evaluated the neurogenesis of neonatal valproic acid (VPA) exposure on subventricular zone progenitors of the developing cerebral cortex in ferrets. VPA was injected at a dose of 200 µg/g of body weight into ferret infants on postnatal days 6 and 7. Two different thymidine analogues, 5-ethynyl-2′-deoxyuridine (EdU) and 5-bromo-2′-deoxyuridine (BrdU), were injected with a 48 h interval to label proliferating cells before and after VPA exposure. Two hours after BrdU injection, BrdU single- and EdU/BrdU double-labeled cells, but not EdU single-labeled cells, were significantly denser in both the inner and outer subventricular zones of VPA-exposed infants than in control infants. Notably, more than 97% of BrdU single- and EdU/BrdU double-labeled cells were immunopositive for Pax6, a stable marker for basal radial glia (bRG), in both groups. In contrast, the percentage of cells positively immunostained for Cux1, a postmitotic marker for upper-layer cortical neurons, in both EdU single- and BrdU single-labeled cells, was significantly higher in VPA-exposed infants than in control infants. These findings suggest that neonatal VPA exposure facilitates bRG proliferation, including self-renewal, followed by their differentiation into upper layer cortical neurons in the premature cortex of ferrets.

## 1. Introduction

Valproic acid (VPA), an antiepileptic/anticonvulsant drug, is also known to be an inhibitor of histone deacetylases 1 and 2 [1]. When various mammalian species [2,3,4,5,6,7,8], including ferrets [9], are exposed to VPA during prenatal and early postnatal development, it may cause social behaviors associated with autism spectrum disorder (ASD). Altered brain morphology due to developmental VPA exposure has been reported mainly in the cerebral cortex, and includes cortical thickening [10,11] or thinning [4], an increased number of cortical neurons [7] and altered gyrification [12,13,14,15]. Our recent study revealed altered sulcal infolding with an increased neuron density and cortical thickening in the sulcal floors following neonatal VPA exposure in a gyrencephalic animal model, the ferret [16]. Some human ASD patients reportedly exhibit similar cortical characteristics [17,18,19].

VPA mediates neurogenesis by regulating the proliferation, maintenance, and differentiation of neuronal stem/progenitor cells, although the effects of VPA vary depending on the exposure time points and neuronal stem/progenitor cell types. For example, VPA mediates the upregulation of Wnt/β-catenin pathways [20] and the proliferation of dentate gyrus (DG) progenitors in the developing hippocampus [21,22], and promotes the adult neurogenesis of DG progenitors by inhibiting HDAC activity [23]. In contrast, VPA alters the cell-cycle exit of subventricular zone (SVZ) progenitors via the inhibition of HDAC activity during the early stage of cortical neurogenesis in mice [11]. In our recent study, VPA exposure during the late stage of cortical neurogenesis increased the density of cortical neurons derived from basal radial glia (bRG) in ferrets [16]. The bRG, also called the outer RG or transient RG, are self-renewable neuronal stem cells that appear transiently in the inner and outer subventricular zones (iSVZ and oSVZ) during cortical neurogenesis in humans [24,25] and other mammalian species [26,27,28,29], including ferrets [30]. They are distinct from intermediate progenitor cells which are classically known cortical progenitors [24,25]. Likewise, bRG emerges in the developing cortex of mice, but is less abundant than in primates and carnivores [26,27]. Therefore, ferrets are more suitable than rodents for investigating the effect of VPA with bRG as a target. Therefore, this study aimed to determine the intermediate influence of VPA exposure on the proliferation, maintenance, and/or differentiation of bRG in the developing cerebral cortex, which may be involved in gyrification anomalies previously reported in ferrets [16]. Two thymidine analogues, 5-ethynyl-2′-deoxyuridine (EdU) and 5-bromo-2′-deoxyuridine (BrdU), were administered within a 48 h interval to label proliferating cells before and after VPA exposure. This administration schedule can also label self-renewing bRG in ferrets [30].

## 2. Results

### 2.1. Immunofluorescence Staining for Various Markers with EdU and BrdU Labeling

**Figure 1:**
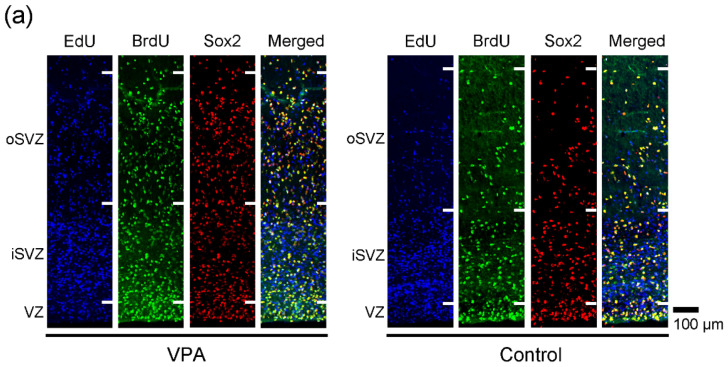

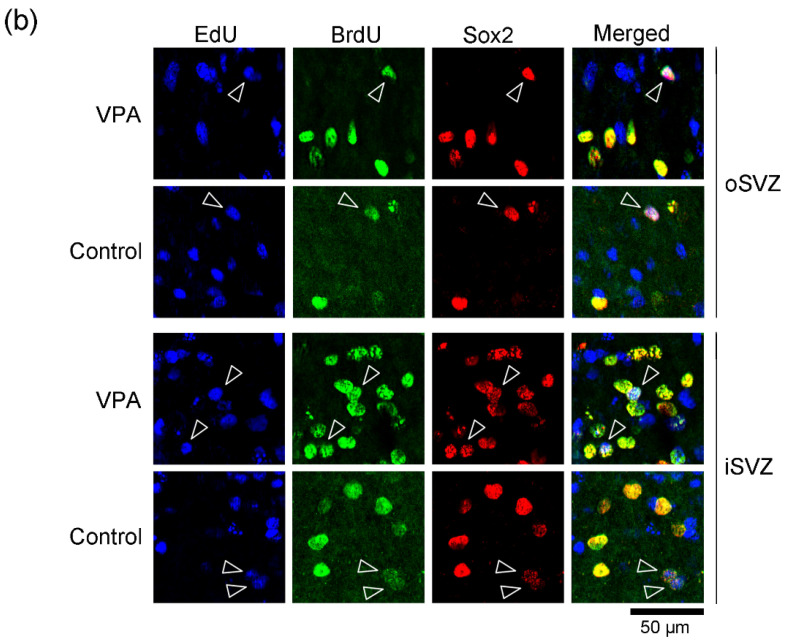
Sox2 immunofluorescence with EdU and BrdU labeling in the SVZ of the premature cortex of VPA-exposed and control ferrets at PD 7. (**a**) Low-magnification images of the oSVZ through iSVZ. (**b**) High-magnification images of the oSVZ and iSVZ. Open arrowheads, Sox2-positive progenitors with EdU/BrdU double labeling.

**Figure 2:**
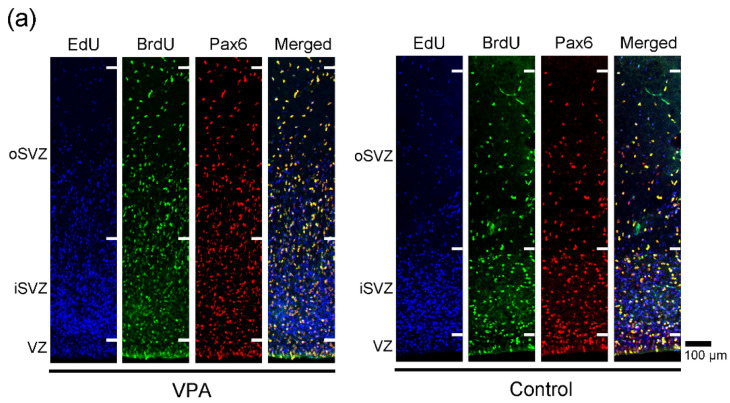

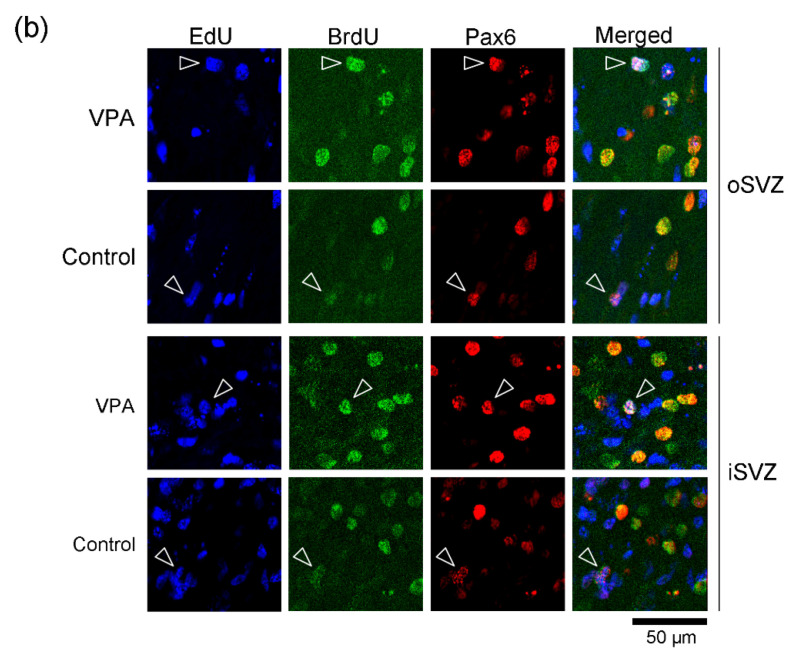
Pax6 immunofluorescence with EdU and BrdU labeling in the SVZ of the premature cortex of VPA-exposed and control ferrets at PD 7. (**a**) Low-magnification images of the oSVZ through iSVZ. (**b**) High-magnification images of the oSVZ and iSVZ. Open arrowheads, Pax6-positive progenitors with EdU/BrdU double labeling.

**Figure 3:**
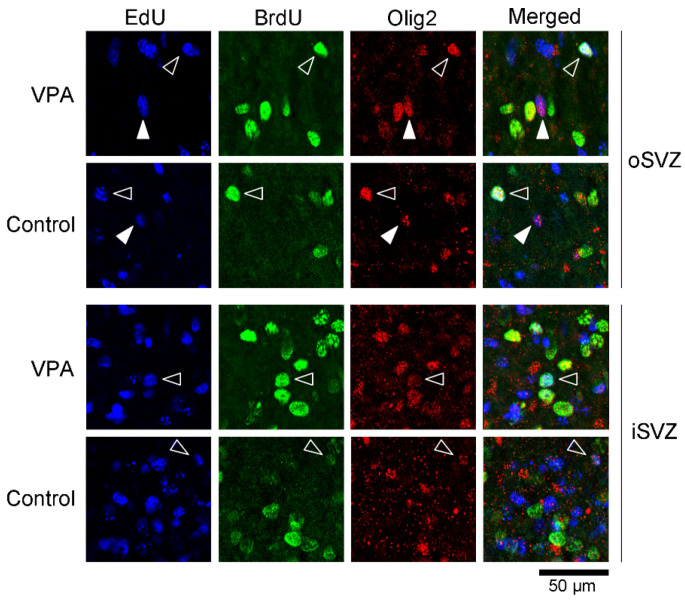
High-magnification images of Olig2 immunofluorescence with EdU and BrdU labeling in the SVZ of the premature cortex of VPA-exposed and control ferrets at PD 7. Open arrowheads, Olig2-positive progenitors with EdU/BrdU double labeling; closed arrowheads, Olig2-positive progenitors with EdU single labeling.

**Figure 4:**
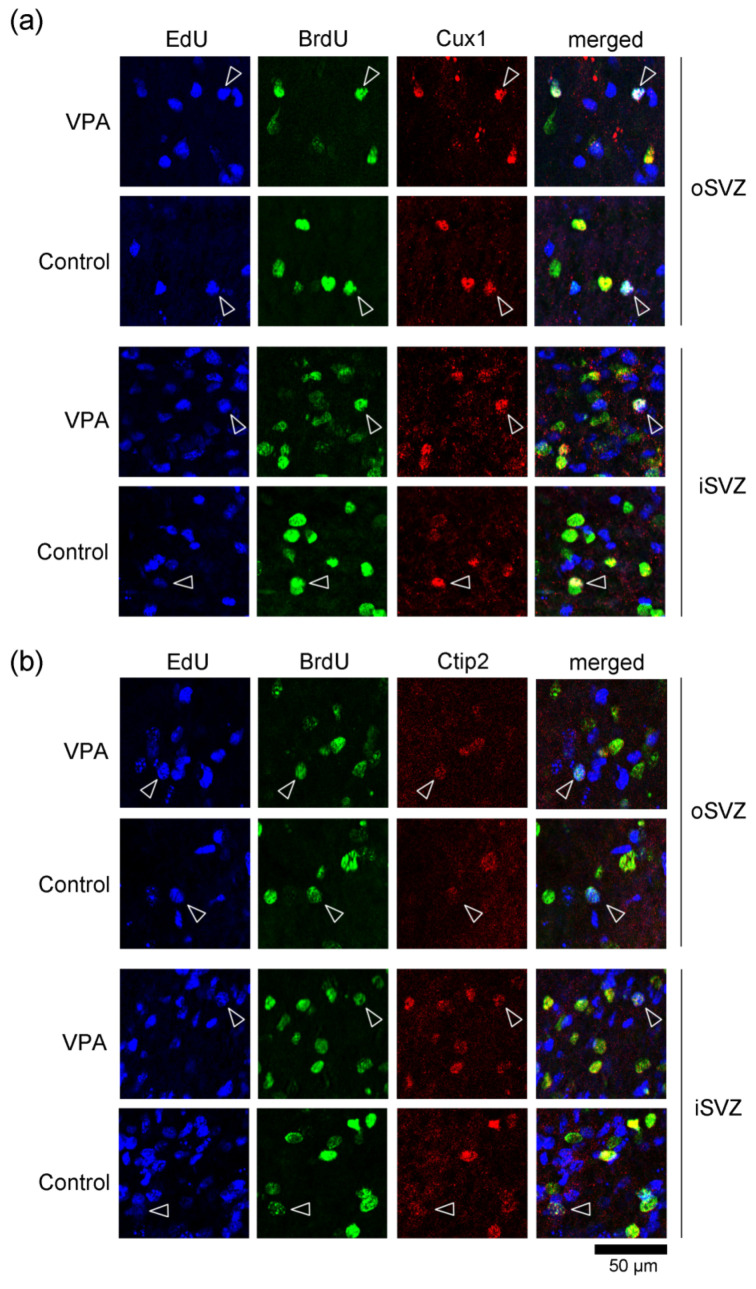
Immunofluorescence for postmitotic markers with EdU and BrdU labeling in the SVZ of the premature cortex of VPA-exposed and control ferrets at PD 7. (**a**) High-magnification images of Cux1 immunofluorescence with EdU and BrdU labeling in the oSVZ and iSVZ. Open arrowheads, Cux1-positive immature neurons with EdU/BrdU double labeling. (**b**) High-magnification images of Ctip2 immunofluorescence with EdU and BrdU labeling in the oSVZ and iSVZ. Open arrowheads, Ctip2-positive immature neurons with EdU/BrdU double labeling.

EdU-labeled cells, which proliferated 24 h before the first VPA injection (at postnatal day (PD) 5), were distributed abundantly in the iSVZ and diffusely in the oSVZ on PD 7 (Figure 1a). Although a great majority of these cells were single-labeled with EdU, some were also labeled with BrdU, indicating that these cells experienced a second round of cell division 48 h after EdU injection in both VPA-exposed and control groups (Figure 1). In contrast, BrdU single-labeled cells were distributed diffusely in the iSVZ through the oSVZ (Figure 1a). BrdU labeling was observed in proliferating cells immediately following the second VPA injection in the VPA-exposed group. Positivity of immunostaining for various markers was examined in EdU single-, BrdU single-, and EdU/BrdU double-labeled cells. Some of these cells were immunopositive for Sox2 (a marker for neuronal stem cells [28,29,31]) (Figure 1); Pax6 (a stable marker for bRG across mammals [28,29,31]) (Figure 2); Olig2 (expressed in progenitors with glial progeny [29,31]) (Figure 3); Cux1 (a marker for postmitotic upper cortical layer neurons [32]) (Figure 4a); and Ctip2 (a marker for postmitotic upper cortical layer neurons [33]) (Figure 4b).

### 2.2. Densities of EdU Single-, BrdU Single- and EdU/BrdU Double-Labeled Cells

**Figure 5:**
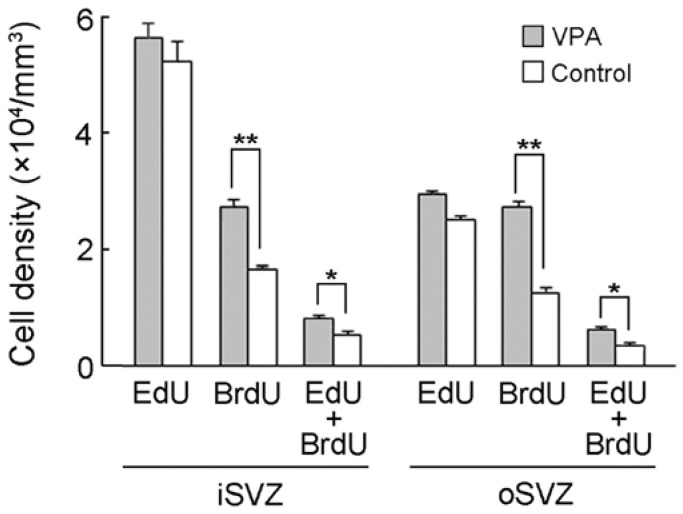
Densities of EdU single-, BrdU single-, and EdU/BrdU double-labeled cells in the iSVZ and oSVZ of the premature cortex of postnatal day (PD) 7 ferrets. Data are shown as mean ± standard error of the mean (SEM). Significance is indicated using Scheffe’s test at * *p* < 0.01, ** *p* < 0.001; number of ferrets = 3 each.

The present study was designed to label proliferating cells with EdU 24 h prior to VPA injection (PD 5) and with BrdU immediately following two consecutive days of VPA injection (PD 7). The 48 h interval between EdU and BrdU administration would lead to the labeling of self-renewing bRG in the ferret premature cortex because it covers the minimum time for the S-shape of either the first or second round of cell divisions [30]. No statistical difference in the density of EdU single-labeled cells between VPA-exposed and control ferret infants was observed for either the iSVZ or oSVZ, indicating no alteration in cell proliferation (Figure 5). In contrast, a significant main effect at the group level was detected in the density of BrdU single-labeled cells by two-way repeated-measures ANOVA (F_(1,__4)_ = 150.928; *p* < 0.001). Scheffe’s test indicated significantly denser BrdU single-labeled cells in both the iSVZ (*p* < 0.001) and oSVZ (*p* < 0.001) in VPA-exposed infants than in control infants (Figure 5). Notably, although a small population of proliferating cells on PD 5 experienced self-renewal after a 48 h interval (EdU/BrdU double-labeled cells), they were significantly denser in VPA-exposed infants than in control infants and were detected either in the iSVZ (*p* < 0.01) or oSVZ (*p* < 0.01) (Scheffe’s test), following a significant main effect on the group by two-way repeated-measures ANOVA (F_(1,__4)_ = 20.196; *p* < 0.05) (Figure 5).

### 2.3. Incidence of Immunostaining for Various Markers in EdU Single-, BrdU Single- and EdU/BrdU Double-Labeled Cells

The incidence of cells immunostained for various markers was estimated in EdU single-, BrdU single-, and EdU/BrdU double-labeled cells in the SVZ, and the results are shown in Table 1. Incidence was estimated independently for each marker antigen. Therefore, the proportion at which the expression of each antigen overlapped with one another is unclear.

In both VPA-exposed and control infants, EdU single-labeled cells were immunopositive for Sox2, Cux1, and Ctip2 in a small population of less than 10% of cells for each marker. Significantly greater incidences of Sox2 (*p* < 0.01) and Cux1 (*p* < 0.01) expression in EdU single-labeled cells were observed in VPA-exposed infants than in control infants (Table 1). Pax6 immunostaining was observed in EdU single-labeled cells at a relatively larger proportion compared with other markers. Pax6 immunostaining was observed in 32.0% of EdU single-labeled cells in control infants, which was significantly reduced to 24.5% in VPA-exposed infants (*p* < 0.05) (Table 1). In contrast, a significant difference in the incidence of Olig2 immunostaining was observed: 14.3% of EdU single-labeled cells in control infants and 6.9% in VPA-exposed infants (*p* < 0.001) (Table 1).

More than 80% of BrdU single-labeled cells were immunopositive for the neuronal stem/progenitor cell markers Sox2 and Pax6 in both groups (Table 1). A significantly greater incidence of immunostaining for Pax6, but not Sox2, was detected in the VPA-exposed infants than in the control infants (*p* < 0.05) (Table 1). Cux1 immunostaining was observed in 24.4% of BrdU single-labeled cells in control infants, and a significant increase (*p* < 0.05) in 34.8% of such cells in VPA-exposed infants (Table 1). There were no differences in the incidence of Olig2 and Ctip2 expression between the two groups (Table 1).

Among EdU/BrdU double-labeled cells, the incidence of Pax6 immunostaining was significantly higher in VPA-exposed infants (98.5%) than in control infants (82.0%) (*p* < 0.05) (Table 1). However, the incidence of Sox2 immunostaining was significantly lower in VPA-exposed infants (86.1%) than in control infants (100%) (*p* < 0.05) (Table 1).

## 3. Discussion

VPA is known to mediate neurogenesis by regulating the proliferation, maintenance, and/or differentiation of neuronal stem/progenitor cells, although VPA exhibits diverse effects depending on the type of neuronal stem/progenitor cells and the developmental stages of the brain [11,20,21,22,23,34,35]. In the current study, more than 90% of BrdU single-labeled cells in the SVZ were immunopositive for Pax6, which is a stable marker for bRG across mammals [28,29,31], and their density increased immediately following VPA exposure in PD 7 ferrets. Such VPA-related changes may not be accompanied by a massive loss of SVZ progenitors, because VPA prevented apoptosis [36] and increased the anti-apoptotic factors (such as Bcl-2) of neuronal progenitors [37,38]. The present study further examined the effect of VPA on SVZ progenitors that had already experienced the S-phase before VPA exposure (on PD 5) using EdU labeling. A large population of proliferating SVZ progenitors on PD 5 was EdU single-labeled, and the density of these cells was not altered by VPA exposure. On the other hand, a small population of proliferating SVZ progenitors on PD 5 was EdU/BrdU double-labeled, and 82.0% of them were Pax6 immunopositive in control infants. Notably, VPA exposure increased EdU/BrdU double-labeled cell density and the incidence of Pax6 immunostaining (98.5%) on PD 7. The 48 h interval between EdU and BrdU administrations applied in the present study covered the minimum times for the S-shape of either the first or second round of bRG cell divisions, allowing self-renewing bRG to be labeled in the ferret premature cortex [30]. Thus, VPA may primarily facilitate SVZ progenitor proliferation, including the self-renewal of bRG, in the ferret premature cortex during the late stage of cortical neurogenesis.

VPA exposure to mice throughout gestation primarily increased the number of Cux1-immunopositive upper cortical-layer neurons and enhanced the non-specific expression of G1-phase regulatory proteins, namely a differentiation-inducing protein p27Kip1, and the differentiation-inhibitory proteins cdk2, cdk4, and cyclinD1 [11]. Juliandi et al. (2012) [34] also reported that Cux1-immunopositive upper layer cortical neurons were induced in vitro from mouse embryonic stem cells by VPA. In the current study, the percentages of cells immunopositive for various markers in SVZ progenitors were significantly altered in ferret infants, although such changes were small. Notably, the incidence of Cux1 immunostaining increased in SVZ progenitors with EdU single and BrdU single labeling immediately after the second VPA injection. These results suggest that VPA exposure at the late stage of cortical neurogenesis also facilitates the differentiation of SVZ progenitors, including bRG, into upper layer cortical neurons in the premature cortex of ferrets. This VPA effect may be mediated by the non-specific enhanced expression of G1-phase regulatory proteins, as observed in mice [11]. Furthermore, the effect of VPA exposure on SVZ progenitors differed before and after S-phase. The expression ratios of various markers in EdU single-labeled cells revealed that SVZ progenitors exposed to VPA after undergoing the S-phase prevented the differentiation into Olig2-positive glial cells and sustained Sox2-positive stem cell characteristics (rather than Pax6-positive bRG characteristics). 

Neonatal exposure to VPA, which covers the late stage of cortical neurogenesis, induces ASD-like social behavioral deficits in ferrets [9]. Our recent study revealed a gyrification abnormality with increased neuron density and cortical thickening in the sulcal floors following neonatal VPA exposure in ferrets [16]. In the current investigation, bRG proliferation (including self-renewal), followed by Cux1 immunostaining, was facilitated immediately following VPA exposure in the premature cortex of ferrets. The findings, therefore, suggest that the neurogenesis of upper-layer cortical neurons from bRG is substantial as VPA effects cause gyrification abnormalities seen in VPA-exposed ferrets.

## 4. Materials and Methods

### 4.1. Animals

Six male ferrets were purchased on PD 5 from Japan SLC (Hamamatsu, Japan). They were reared with lactating ferret dams (3 pups/mother) in stainless-steel cages (80 cm × 50 cm × 35 cm) kept at 21.5 °C ± 2.5 °C under 12 h artificial illumination in the Facility of Animal Breeding, Nakaizu Laboratory, Japan SLC. All dams were fed a pellet diet (High Density Ferret Diet 5L14, PMI Feeds, Inc., St. Louis, MO, USA) and provided tap water ad libitum.

All ferret infants were injected intraperitoneally with EdU at 30 µg/g body weight (Sigma-Aldrich, St. Louis, MO, USA) on PD 5 and BrdU at 30 µg/g body weight (Sigma-Aldrich) on PD 7. Three infants were administered VPA intraperitoneally at 200 µg/g body weight on PD 6 and 7, corresponding to the late stage of cortical neurogenesis [31]. The schedule for VPA administration was designed in accordance with our recent study [16]. The second injection of VPA was administered at the same time as the BrdU injection. The remaining three infants that did not receive VPA were used as controls. Two hours after BrdU injection, all infants were perfused with 4% paraformaldehyde (PFA) in PBS under deep anesthesia with ~2% isoflurane gas.

### 4.2. Immunofluorescence Procedures

The cerebral hemispheres were separated on the left and right sides of the longitudinal cerebral fissure, immersed in 30% sucrose in PBS, and embedded in an optimal cutting-temperature compound. Coronal sections were made at 100 μm thickness using a Retratome (REM-700, Yamato Koki Industrial Co., Ltd., Asaka, Japan) with a refrigeration unit (Electro Freeze MC-802A, Yamato Koki Industrial). The brain tissue of infant ferrets is very soft due to unmyelination and a high water content. To maintain the integrity and morphology of the premature cortex, it was necessary to make cryosections of 100 μm thickness. All sections were collected in vials containing a 4% PFA solution. 

Five serial coronal sections taken at the level of the anterior commissure underwent immunofluorescence staining and EdU detection. These sections included large expansions of both the iSVZ and oSVZ, and the cortical region examined corresponded to the primary somatosensory cortex [39]. All procedures were performed on floating sections, according to a previous report [30]. Sections were heated in Antigen Retrieval Reagent UNIVERSAL (R&D system, Minneapolis, MN, USA) for 30 min in a 90 °C water bath and then cooled at 4 °C for 30 min. Two hours following a preincubation with PBS containing 0.1% Triton-X 100 (Triton-PBS) at room temperature at 37 °C, EdU was detected on the sections by a reaction with a Click-iT reaction cocktail containing Alexa Fluor 488 (Click-iT EdU Alexa Fluor 488 Imaging Kit, Thermo Fisher Scientific, Waltham, MA, USA) at 37 °C for 2 h. Then, sections were incubated with a mixture of a rat BrdU monoclonal antibody (1:1000; ab6326, Abcam, Cambridge, UK), a goat Sox2 polyclonal antibody (1:500; AF2018, R&D Systems, Minneapolis, MN, USA), mouse Pax6 monoclonal antibody (1:500, ab78545, Abcam, Cambridge, UK), mouse Cux1 monoclonal antibody (1:500, SC-514008, Santa Cruz Biotechnology, Inc., Santa Cruz, CA, USA), rat Ctip2 monoclonal antibody (1:500, ab18465, Abcam), or rabbit Olig2 polyclonal antibody (1:500; IBL, Gunma, Japan) dissolved in Triton-PBS containing 10% normal horse serum (Vector Labs. Inc. Burlingame, CA, USA) at 4 °C overnight. Highly specific immunostaining was reported in the ferret brain tissue using these primary antibodies [30,40,41]. The sections were subsequently incubated at 37 °C for 2 h with appropriate secondary antibodies, which included Alexa 350 goat anti-rat IgG (1:500; A-21093; Thermo Fisher Scientific, Waltham, MA, USA), Alexa 350 donkey anti-sheep IgG (1:500; A21097, Abcam), Alexa 647 chicken anti-rat IgG (1:500; A-21472, Thermo Fisher Scientific), biotinylated horse anti-mouse IgG (1:200; BA-2001, Vector Labs), and biotinylated donkey anti-rabbit IgG (1:200; A16027, Thermo Fisher Scientific). When using biotinylated secondary antibodies, sections were further incubated with Alexa 555-conjugated streptavidin (1:500, S21381, Thermo Fisher Scientific) at 37 °C for 1 h.

### 4.3. Evaluating the Density of Immunostained and/or Thymidine Analogue-Labeled Cells

Serial digital sectioning images were acquired at a 10 μm depth (section plane thickness = 1 μm; number of sections = 10) from the most superficial plane, where EdU and BrdU labeling with immunostaining for various markers were obtained. Images were captured with a 20x objective using an Axio Imager M2 ApoTome.2 microscope with a 20× objective equipped with an AxioCam MRm camera (Zeiss, Gottingen, Germany) with Zen 2.3 blue edition software (Zeiss). A set of sectional images, 4 μm apart in the Z-direction (the third and seventh from the superficial slices of the acquired images), were selected as the lookup and reference images, respectively. The disector method using systematic random sampling was used to estimate the density of immunostained and EdU- and/or BrdU-labeled cells according to a previous report [30]. In sections immunostained for each marker, frames with 12 square boxes (box size = 40 × 40 μm) were used from one section (the left or right hemispheres) to systematically select the region of interest (ROI) randomly superimposed on the iSVZ and oSVZ of both the lookup and reference images at the same positions perpendicular to the ventricular surface. Thymidine analog-labeled or immunostained cells were counted within the ROIs using the “forbidden line” rule [42]. Their densities were calculated using the following formula: [Cell density = Qn−/(a × b × t)] (Qn− = total number of thymidine analogue-labeled and/or immunostained cells appearing within ROIs in the lookup images, but not in the reference images; a = 24, total number of ROIs in the lookup images from two sections (the left and right hemispheres) per animal; b = 40 × 40 μm, areas of counting box; and t = 4 μm, distance between the lookup and reference images). 

The percentages of immunostained cells and EdU- and/or BrdU-labeled cells were estimated by summing the cells counted within all ROIs from all animals of each group.

### 4.4. Statistical Analysis

Measurements from the left and right hemispheres were combined and the number of animals (n) was set to “3” in each group. The density of immunostained cells and EdU- and/or BrdU-labeled cells were statistically analyzed by two-way repeated-measures ANOVA with the region (iSVZ and oSVZ) and group (VPA-exposed and control groups) as factors. For post hoc testing, Scheffe’s test was applied to detect significant differences in the group and/or region × group interactions using two-way repeated-measures ANOVA and simple main effects.

The percentage of cells immunolabeled for the markers among thymidine analogue-labeled cells was statistically assessed using the χ^2^ test. The total number of EdU single-, BrdU single-, and EdU/BrdU double-labeled cells was defined as “n” for the χ^2^ test.

## 5. Conclusions

VPA mediates neurogenesis [11,20,21,22,23,34,35]. It alters the cell-cycle exit of SVZ progenitors (largely the intermediate progenitor cells) during the early stage of cortical neurogenesis in mice [11]. Similar results were obtained in this ferret model, but findings on the stage of neurogenesis and the SVZ progenitor type altered by VPA were distinct. Notable findings of this study are that VPA exposure facilitated the proliferation of SVZ progenitors, mainly Pax6-positive bRG, and their differentiation into Cux1-positive upper layer cortical neurons. The bRG are a major source of a massive expansion of the cerebral cortex, particularly in gyrencephalic mammals [43], and bRG-derived neurons were placed densely in multimodal-associated cortical regions [30]. Thus, a ferret model can offer new insights into the pathogenesis of neurodevelopmental disorders affecting cortical neurogenesis caused by epigenetic factors such as VPA, which may not be observed in a mouse model.

## Figures and Tables

**Table 1:** Incidence of immunostained cells for various markers in EdU single-, BrdU single- and EdU/BrdU double-labeled cells in the subventricular zone of the premature cortex.

	VPA		Control	
EdU+ cells				
% of Sox2+	7.8%	(25/320) **	2.5%	(8/315)
% of Pax6+	24.5%	(91/372) *	32.0%	(88/275)
% of Olig2+	6.9%	(22/320) ***	14.3%	(45/315)
% of Cux1+	5.8%	(25/430) **	1.8%	(7/369)
% of Ctip2+	4.7%	(20/430)	2.3%	(9/396)
BrdU+ cells				
% of Sox2+	83.7%	(154/184)	88.8%	(87/98)
% of Pax6+	97.6%	(201/206) *	92.5%	(99/107)
% of Olig2+	26.1%	(48/184)	34.7%	(34/98)
% of Cux1+	34.8%	(106/305) *	24.4%	(41/168)
% of Ctip2+	41.6%	(127/205)	37.5%	(63/168)
EdU+/BrdU+ cells				
% of Sox2+	86.1%	(31/36) *	100%	(26/26)
% of Pax6+	98.5%	(66/67) *	82.0%	(41/50)
% of Olig2+	41.7%	(15/36)	26.9%	(7/26)
% of Cux1+	31.8%	(21/66)	16.7%	(5/30)
% of Ctip2+	30.3%	(20/66)	23.3%	(7/30)

Percentages were calculated by summing each labeled cell counted within all ROIs from the inner and outer subventricular zones in the premature cortex from 3 ferrets. The numbers of labeled cells used to calculate the percentages are shown in parentheses. * *p* < 0.05, ** *p* < 0.01, *** *p* < 0.001 vs. control (χ^2^ test). EdU+, EdU single-labeled; BrdU+, BrdU single-labeled; EdU+/BrdU+, EdU/BrdU double-labeled; Sox2+, Sox2 immunostaining; Pax6+, Pax6 immunostaining; Olig2+, Olig2 immunostaining; Cux1+, Cux1 immunostaining; Ctip2+, Ctip2 immunostaining.
